# What determines the performance of small and medium-sized enterprises supply chain financing? A qualitative comparative analysis of fuzzy sets based on the technology–organization–environment framework

**DOI:** 10.3389/fpsyg.2022.976218

**Published:** 2022-11-17

**Authors:** Weichang Duan, Hanzhou Hu, Yuting Zhang

**Affiliations:** ^1^School of Management, Guangzhou University, Guangzhou, China; ^2^School of Business Administration, Anhui University of Finance and Economics, Bengbu, China

**Keywords:** supply chain financing, TOE, fsQCA, medium-sized and small enterprises, environmental competitiveness

## Abstract

With the COVID-19 pandemic sweeping the globe, small and medium-sized enterprises’ (SMEs’) survival space has been increasingly constrained, and their financing challenges and costly concerns have also become more evident. With the emergence of the supply chain financing model, the problem of difficult financing for SMEs has been effectively alleviated. How to effectively improve the performance of supply chain financing for SMEs is a hot issue of common concern for both business and academic circles. This paper used 90 SMEs involved in supply chain financing business for a case study based on the “Technology–Organization–Environment” (TOE) framework, using fuzzy set qualitative comparative analysis (fsQCA), and explored the linkage effects of technology, organization, and environmental conditions on SMEs’ performance in improving supply chain financing and their path choices. The study found that: (1) individual conditions do not constitute a necessary condition for high/low supply chain financing performance. However, technical level preconditions play a more important role in shaping the high supply chain financing performance of firms. (2) The three preconditions at the technological, organizational, and environmental levels work together to form a diverse set of conditions that drive the high supply chain financing performance of firms. That is, the paths driving the high supply chain financing performance of SMEs are characterized by “different routes to the same destination.” There are three different models: “technology-supply chain capability-driven,” “IT-supply chain capability-driven,” and “IS-supply chain capability-driven.” (3) The degree of application of corporate information technology, information sharing capability, and supply chain capability, and the lack of environmental competitiveness are the reasons for the generation of the low supply chain financing performance of SMEs. The above research findings can provide a direct theoretical basis for enterprise supply chain financing practice and are of great practical significance in guiding Chinese SMEs on how to improve their supply chain financing performance.

## Introduction

China’s economic development has been greatly hampered by the new coronavirus outbreak. According to public data from the National Bureau of Statistics, private enterprises achieved a total profit of 120.83 billion CNY in January–February 2020, down 36.6% year on year due to the impact of the epidemic. During the epidemic, the weaknesses of SMEs and their dependence on cash flow gradually became apparent, causing them to face shortages of working capital and breaks in cash flow (Qin, 2021). SMEs are an integral part of our economic map. SMEs play an important role in providing employment for our population and in regulating the structure of the national economy (Xing, 2018). However, enterprises in development often encounter the problem of capital shortages, which has seriously restricted the rapid development of SMEs. SMEs are limited by their size, lack of collateral assets, lack of credit, and other factors, resulting in banks and other financial institutions being reluctant to lend to them due to risk control considerations.

Academics have conducted active research on how to help SMEs solve their financing dilemmas. Among them, the supply chain financing model is unanimously recognized by most scholars, who believe that this model can solve the financing problems of SMEs (Xin, 2007; Zhao, 2020; Pan et al., 2021). Supply chain finance has significant advantages in reducing transaction costs, weakening information asymmetry, and enhancing risk control, and plays an important role in solving the problem of difficult financing for SMEs (Xia and Jin, 2011). This financing model focuses on the authenticity and continuity of the transaction background, the closed nature of the business operation, and the self-repayment of the loan. Its main role is to solve the problem of financing difficulties caused by the lack of credit and collateral assets of SMEs, and to obtain funds from banks and other financial institutions with the credit of core enterprises to support their own development. The research on supply chain financing has found that not every supply chain enterprise can make use of the credit support obtained by the supply chain financing model, even if they are in the same supply chain. It is of strong practical guidance to explore those conditions that can affect the supply chain financing performance of enterprises, so that SMEs in the supply chain can build on their strengths and avoid their weaknesses to better obtain financial support from financial institutions.

As the research on supply chain financing continues, it is found that even for enterprises in the same supply chain, not every supply chain enterprise can make use of the credit support obtained by the supply chain financing model (Wei and Liu, 2012; Song et al., 2017a). In order to help SMEs better access supply chain financing and relieve their financial pressure, it is necessary to further dissect the mechanisms affecting the performance of supply chain financing for SMEs. In reality, the supply chain financing performance of SMEs is influenced by many factors, which are inevitably interrelated and work together to form a high supply chain financing performance. However, considering the limited number of previous research perspectives and analytical frameworks, it is difficult to deeply elaborate on the mechanisms affecting the supply chain financing performance of SMEs, leading to difficulties in determining the driving paths to improve the supply chain financing performance of SMEs. Thus, the research question of this paper is posed: what are the factors that affect the supply chain financing performance of firms? And how will these factors work together to affect the firm’s supply chain financing performance?

Most of the existing studies have explored the relationship between individual factors such as firm network embeddedness and improved innovation capacity and supply chain financing performance. Based on principal-agent theory, found that intra-firm financial collaboration and financial collaboration between buyers and suppliers have a significant contribution to supply chain financing performance (Wandfluh et al., 2016). Combining information asymmetry theory and network theory, some scholars suggest that strong and bridge connections in supply chain networks can effectively promote the quality of financing for SMEs. Martin (2017), on the other hand, sorted out the role of supplier dependence, buyer bargaining power, and buyer–supplier trust in facilitating supply chain financing based on social exchange theory. Martin and Hofmann (2019) proposed based on transaction cost theory through an exploratory multi-case analysis that the effectiveness of supply chain financing can be judged based on two dimensions: the timing of financing and the source of funds. In addition, other scholars have explored the mechanism of the intrinsic influence of a firm’s internal and external capabilities on supply chain financing performance based on capability theory using supply chain financing solution adoption as a mediating variable. These studies are mostly a power variable perspective, exploring the linear relationship between a single factor or moderating variable on firms’ supply chain financing performance, which limits the understanding of the synergistic matching effects among multiple factors—such as technology, organization, and environment—behind the differences in SMEs’ supply chain financing performance.

To address the shortcomings of the above study (Wei and Liu, 2012; Wandfluh et al., 2016; Martin, 2017; Martin and Hofmann, 2019), this paper finds that the key point for SMEs to obtain supply chain financing is the willingness of the core enterprises in the supply chain to guarantee for them through the analysis of the supply chain financing model. In this paper, according to the logic that the characteristics of SMEs affect the willingness of core enterprises to guarantee their supply chain financing performance, five factors affecting the performance of supply chain financing are selected from the three levels of “technology, capability, and environment” to build a theoretical model framework affecting the performance of supply chain financing of SMEs. The fuzzy set qualitative comparative analysis (fsQCA) method is used to analyze the grouping of these five factors and derive the conditional grouping and the mechanism of action that cause the difference of enterprise supply chain financing performance; then, by exploring the linkage effect of many factors that affect performance, it helps to establish a systematic analysis idea for constructing the development path of SME supply chain financing performance. Secondly, it enables SMEs in the supply chain to build on their strengths and avoid their weaknesses, increasing the willingness of core enterprises to guarantee for them, and thus better access to financial institutions’ financial support.

## Literature review and research framework

### Application of fsQCA method in the field of supply chain finance

In order to explore the impact mechanism of blockchain technology on supply chain finance, Hong et al. (2022) established the basic theoretical model of “digital credit co-governance-network embeddedness-supply chain finance performance” from the perspective of network embeddedness, used partial least squares to verify the structural equation model, and further used fuzzy set qualitative comparison analysis to analyze four financing performance dimensions, namely financing cycle, financing amount, financing availability, and financing cost, as the outcome variables for group analysis. Based on the basic theoretical logic of “firm capability-competitive advantage—firm performance” in the theory of firm capability, Lu et al. (2019a) uses fuzzy set qualitative comparative analysis to explore the impact of SMEs’ innovation capability and market responsiveness as well as the adoption of supply chain financing solutions on the performance of supply chain financing. The impact on supply chain financing performance is explored by using fuzzy set qualitative comparative analysis. Li and Sun (2022) used “financing capability-financing intermediary-financing performance” as the theoretical research framework to construct the cooperative capability of core enterprises, and tested the research hypotheses based on Discriminant Analysis (DA) and Fuzzy set Qualitative Comparison Analysis (Fs-QCA) through the theoretical model that supply chain network integration affects supply chain financing performance. Yu and Wang (2022) used an empirical approach combined with the fuzzy set qualitative fixed ratio analysis (fsQCA) method to investigate the core firms’ willingness to participate in supply chain finance and their financing model orientation.

In summary, the application of fuzzy set qualitative comparative analysis method in the field of supply chain amount has been widely paid attention to, and the advantage of fuzzy set qualitative comparative method which has both qualitative and quantitative analysis in studying the problem of causal complexity is obvious. Most of the existing studies, however, use fsQca as a complement to test the hypothesis.

### Study on influactors of supply chain financing performance

A review of the existing papers reveals that studies on the factors influencing the performance of supply chain financing can be grouped into the following areas:

A lot of theoretical analysis has been described in these papers on how these ITs can be applied in supply chain finance, and specific application architectures have been provided. Didi (2019) suggested that supply chain finance itself is a high-tech business that fits well with blockchain technology, IoT technology, and other information technologies. Further, Yanni (2020) proposed that one of the important reasons for the difficulty of financing SMEs is due to the information asymmetry between banks and enterprises, while big data can solve the problem of information asymmetry, suggesting that SMEs can use big data technology to enhance their financing ability. Most of the existing papers analyze the application of information technology in supply chain financing from a theoretical perspective, which lacks persuasive power. There is little research in the literature that empirically examines the impact of information technology use on the supply chain financing performance of SMEs.

Some scholars have considered the impact of the characteristics of the organizational level of the firm on the performance of supply chain financing. Numerous scholars have studied the impact of firms’ capabilities on supply chain financing performance, including individual entrepreneurs’ capabilities, firms’ core competencies, and firms’ supply chain capabilities. Song et al. (2017b) research found that a firm’s distribution operations capability and demand management capability have a significant positive effect on financing performance. These characteristics of a company’s capabilities are not easily perceived by the financing provider during the SME financing process and are not as intuitive as reviewing the “hard information” of the company. Scholars have concluded that there is a significant correlation between firm size and the number of years in business and financing performance. For example, the study by Petersen and Rajan (1997) concluded that the longer the enterprise is established, the closer the connection between the enterprise and upstream and downstream enterprises in the industry chain, the easier it is to form a cooperative relationship of interest, and the easier it is to get a loan. Zhao (2008), on the other hand, from the perspective of firm size, suggested that the larger the company is, the more collateral it has for its assets when facing business risks; further, its solvency will be stronger than that of smaller companies, and the core companies will have fewer concerns when making credit guarantees for them. As a result, larger companies are more reliable to financial institutions in terms of business credit compared to smaller companies.

Some scholars have also included firm-environment-level characteristics in their analysis of supply chain financing performance. In this paper, we focus on SMEs in the supply chain, and refer to their interactions with other firms in the supply chain as the internal supply chain environment and to those outside the entire supply chain as the external environment. From the perspective of supply chain networks, some scholars study the impact of factors such as the health of supply chain networks, the embeddedness of supply chain networks, and the strong and weak connections between supply chain networks on financing performance. Chen and Song (2020) argue that SMEs joining a healthy business network helps SMEs to improve their dual competence, which in turn improves their position in the network and ultimately contributes to their financing performance. Regarding the influence of the external environment on financing performance, scholars mainly consider factors such as external financing policies as well as the external financing environment. Li and Wang (2017), on the other hand, found that the external environment did not have a significant effect on access to Internet supply chain financing for micro and small enterprises.

### Construction of a supply chain financing performance model for SMEs

**Figure 1:**
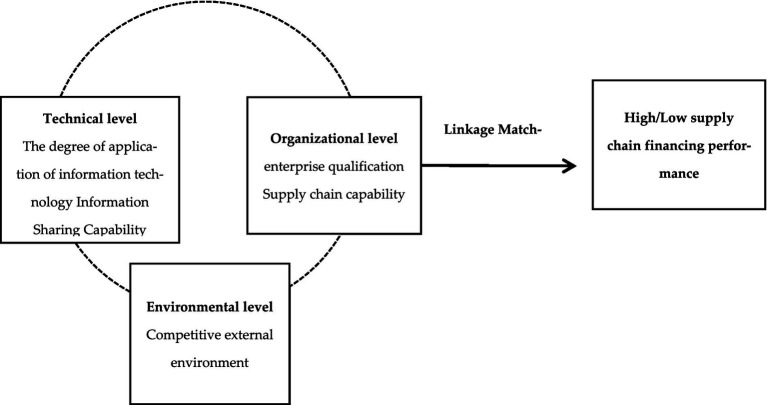
Research model.

Based on the Technology–Organization–Environment (TOE) analysis framework, this paper constructs a theoretical model framework, which analyzes the supply chain financing performance of SMEs by combining the external environmental characteristics of SMEs with the internal characteristics of firms, as shown in Figure 1.

#### Technical level

With the “Internet +” background, a new generation of information technology continues to emerge, such as blockchain technology, big data technology, and the Internet of Things (IoT). The use of information technology not only enhances the management of SMEs themselves and improves their efficiency in production, but also plays a vital role in reducing information asymmetry. This increases the level of trust of core enterprises, making them more willing to provide credit guarantees for SMEs, which ultimately improves the availability of supply chain financing for SMEs.

The model specifically includes the degree of information technology application and enterprise information sharing capabilities, which are two secondary conditions. In supply chain management, information technology is a general term for all the technologies used in management as well as information processing (Zhou and Wang, 2016). In business management, the application of information technology usually takes the form of various information systems, such as MRP, ERP, Logistics Management Systems, etc. The resource-based view considers that a firm’s competitive advantage comes from the use of the resources it has. If companies are able to transform the IT resources they have into the ability to use IT, it will give them a long-term competitive advantage (Bharadwaj, 2000). In addition, the application of information technology to all processes of enterprise products can lead to the effective management of enterprise information flow, logistics, capital flow, and business flow, improving the productivity of enterprises and reducing their operating costs, thus helping them to achieve better business performance. Higher performance will reduce the default risk of companies and can help them obtain supply chain financing at a lower cost.

In addition, the degree of IT application is an important basis for enterprises to obtain information-sharing capabilities. From the perspective of information asymmetry, many scholars believe that the difficulty of financing SMEs is due to the information asymmetry between banks and enterprises. In traditional lending theory, banks and other financial institutions mostly rely on “hard information” such as financial statements uploaded by enterprises when reviewing loans, which makes it difficult to truly understand the business situation of enterprises. The consideration of avoiding a moral hazard leads to banks and other financial institutions being afraid or unwilling to lend. The application of information technology by enterprises can break the information blockage between upstream and downstream enterprises in the supply chain, enabling enterprises to share information across organizational boundaries, while also improving the efficiency of data analysis and processing. Information sharing has a significant direct positive impact on enterprise performance (Yang et al., 2022a). It significantly reduces the degree of information asymmetry between supply chain companies, and improves the information-sharing ability of companies (Kathuria et al., 2018). When external fund providers know more about an enterprise, they are also more willing to provide funds for its development, which in turn increases the possibility of SMEs obtaining supply chain financing.

#### Organization level

##### Enterprise qualification

This level mainly considers enterprise qualifications and enterprise supply chain capability. Enterprise qualifications specifically include the number of employees, the number of years in business, and the annual sales of the enterprise. Akben-Selcuk (Selcuk and Altiok-Yilmaz, 2017) pointed out that it is often the larger firms that have more competitive and more profitable opportunities. According to the theory of enterprise capability, enterprise capability can contribute to the business performance of an enterprise. Similarly, for supply chain financing for SMEs, companies with stronger market competitiveness tend to have higher operating performance, and higher operating performance indicates stronger debt servicing ability. This can bring a higher level of credit to the enterprise, thus reducing the high costs caused by the lack of credit in its financing process and improving the availability of financing to a certain extent. On the other hand, the larger the scale of operation, the longer the operating life, and the larger the annual sales, the more stable the production, supply, and sales capacity of the enterprise, which can support the business activities of other companies in the supply chain. In turn, the company’s position in the supply chain network is enhanced, and it is more easily recognized by the core enterprises and incorporated into their own business network, thus helping them to obtain funds through the use of supply chain financing.

##### Enterprise supply chain capability

Supply chain finance is a financing activity that relies on the supply chain management context, in which the traditional financial attributes are diluted (Caniato et al., 2016; Gelsomino et al., 2016). The most important feature of supply chain financing that is different from traditional enterprise financing is that when banks examine the financing needs of enterprises and decide whether to give them credit support, they do not consider the financial information and other “hard information” of enterprises as the basis for lending, but focus on the capabilities and development prospects of enterprises. In supply chain financing, corporate capacity has become a more important indicator than assets, and it is believed that, the stronger the capacity, the better the development prospects of the company, which tends to also have better solvency. According to signaling theory, SMEs should take the initiative to send signals that reflect the strengths of their enterprises to banks and other financial institutions when financing the supply chain, so that quality SMEs can be identified by the fund providers. Further, the supply chain capacity can reflect the business quality and management level of the companies in the supply chain itself, which is the premise that the funds can be repaid in time (Xiong et al., 2009; Dong et al., 2013). This originates from a series of practices and complex interactions in the supply chain practices of companies (Liu et al., 2016).

The measurement of the supply chain capability of an enterprise mainly considers the distribution operation capability and demand management capability of the enterprise. Some scholars have conducted empirical studies on the relationship between firms’ supply chain capability and financing performance. Song et al. (2017b) found that distribution operational capability and demand management capability have a significant positive effect on financing performance through an empirical study of 150 SMEs’ supply chain finance activities. Distribution operation capability mainly refers to the enterprise’s ability to coordinate and optimize all aspects of product production, processing, and distribution in accordance with the process of supply chain operation, to achieve control and management of the entire business chain, to reduce distribution operation costs, and to achieve basic profitability goals. For banks, a company’s ability to operate in distribution sends a signal that it has stable operating income. Demand management capability mainly refers to the ability of enterprises to respond to customers’ needs. With the improvement of people’s living standards, the demand for goods also shows diversified characteristics. How companies can meet customers’ needs for special goods and customization, and achieve their own profitability goals by creating customer value, are the issues to be considered for companies to gain competitive advantage in the market. In general, companies with demand management capabilities are able to maintain close relationships with their customers and provide the necessary guarantees for long-term profitability. For banks, a firm’s demand management capabilities convey information about higher solvency.

#### Environmental level

The environmental dimension mainly considers the competitiveness of the external environment of the supply chain network. Although environmental-level antecedent conditions do not directly affect corporate supply chain financing performance, in supply chain finance, as it is an activity nested in the supply chain, the influence of the external environment on the supply chain inevitably affects the behavior of the companies in the supply chain. Environmental competitiveness mainly reflects the number of competitors in the external environment as well as the number of competitive fields (Hill and Matusik, 1998).

Previous studies have shown that environmental contestability can significantly contribute to the degree of IT uptake by firms (Gong and Ding, 2014). When the external environment of an enterprise is more competitive, the survival pressure on the enterprise will be greater. In order to obtain a stable market share and maintain its own competitive advantage, the enterprise will be forced to make technological changes to adapt to the strong competitiveness of the external environment. In other words, in a competitive environment, companies will tend to use information technology to improve their information-sharing capabilities and thus avoid problems that hinder the performance of supply chain financing, such as operational instability.

From the perspective of the funding provider, when the external environment becomes more competitive, the funding provider needs to be more aware of the true operational information of the firm and reduce the opportunistic behavior of the firm (Lima et al., 2020). Further, the higher the degree of information technology application, the better the information-sharing ability and the higher the information transparency. This will enable the supply chain capability signal to be more effectively communicated and identified by the capital provider, thus reducing the risk level of the capital provider in providing supply chain financing. This enables companies to obtain supply chain financing with lower financing costs and higher financing efficiency, thus improving their supply chain financing performance.

### Research review

This paper considers that among the available studies on the performance of corporate supply chain financing, there are more in-depth studies and richer conclusions on the connotations, models, performance measurement methods, and influencing factors of supply chain financing. These research results are important guidance and inspiration for this paper, and enrich its theoretical foundation. After combing through the historical literature, the following shortcomings were found in previous studies, which are summarized below.

#### Variable selection

Most scholars who have studied the performance of supply chain financing in the past have considered the influence of the internal factors of enterprises (Lu et al., 2019b; Sun et al., 2021; Zhang, 2021; Yang et al., 2022b), and fewer have considered the influence of the characteristics of the external environments of enterprises on the performance of supply chain financing, and the influence of the combination of the internal factors and external environments of enterprises on the performance of supply chain financing. If studies are detached from the external environment, and do not take into account the influence of the real situation of the competitive nature of the external environment of the enterprise, such studies do not provide practical advice to SMEs in the supply chain on how to access supply chain financing. Paying attention to and studying the external environmental factors of the enterprise will help the enterprise to identify the risks from the market, improve the application of information technology, better form its core competitiveness, and finally obtain supply chain financing solutions to solve their own capital shortage problems.

#### Research perspective and research methodology

Previous studies have mostly been based on a power-variance perspective, constructing theoretical models based on specific research contexts and formulating causal hypotheses (Petersen and Rajan, 1997; Zhao, 2008; Song et al., 2017b; Chen and Song, 2020). To explore the simple linear relationship of an individual factor or moderating variable on a firm’s supply chain financing performance, the study of the synergistic matching effect among multiple factors behind the variation in SME supply chain financing performance is neglected. In addition, there are limitations inherent in traditional research methods, such as regression analysis. The current study could only consider the effects of two or three variables, by setting moderating variables to analyze the interaction term. To observe changes in the direction or the extent of the main effect in various conditions, and less frequently, the joint effect of the simultaneous presence of multiple factors on supply chain financing performance is considered. In the real situation, the difference in supply chain financing performance is the result of multifactor coupling, and research should be closer to the real operating environments of enterprises.

In summary, since the influence of different factors on the performance of corporate supply chain financing is not independent, they will affect the performance of corporate supply chain financing by linkage matching to generate different combinations among them. Therefore, a study with the “grouping perspective” can help deepen the understanding of the complex mechanisms behind the performance of corporate supply chain financing (Tan et al., 2019). Based on the TOE framework, this study selects five antecedent conditions at the “technology–organization–environment” level, and then conducts a comprehensive analysis of the factors affecting the performance of supply chain financing of SMEs. Using fuzzy-set qualitative comparative analysis (fsQCA) to explore the conditional groupings and mechanisms of action that contribute to differences in SME supply chain financing performance, the driving paths that generate high supply chain financing performance is derived. This helps to establish systematic analysis ideas for the development path of improving the supply chain financing performance of SMEs, and enriches the research on supply chain financing performance.

## Research design and data collection

### Variable design

1. Drawing on the scales used by Bruque Camara et al. (2015) and Subramani (2004), the research design emphasized the use of information technology by enterprises for resource acquisition and analysis and in support of business processes. The extent to which information technology was used within enterprises to support business processes, the extent to which enterprises used information technology to share resources with partners in the supply chain network, and the extent to which enterprises used information technology to access organizational resources outside the supply chain network were all analyzed. Three dimensions measured the degree of IT adoption in SMEs, including four specific measurement questions.
2. Enterprise information sharing capability refers to the ease and extent to which financing companies can obtain information about SME operations from the supply chain network. Its measurement was mainly based on the studies of Calanni et al. (2015) and Formentini and Taticchi (2014), among others, and consisted of three specific measurement questions.
3. This paper argues that the level of enterprise qualification can affect the repayment ability and willingness of enterprises, which in turn can affect the willingness of core enterprises in the supply chain to provide credit guarantees for them. The measurement of enterprise qualification was mainly based on four aspects: enterprise size (number of employees), years of operation, annual sales, and total assets of the enterprise.
4. For the measurement of enterprise supply chain capabilities, two indicators were selected, namely, enterprise distribution operation capability and customer demand management capability. Drawing mainly on the study by Zhang et al. (2010), the distribution operation capability was measured in terms of the level of management of raw materials by the company’s internal transportation system, the timeliness of goods delivery, the speed and accuracy of order picking, etc. Specifically, there were six measurement questions. Drawing primarily from Schary and Coakley (1991), the company’s ability to respond to customer needs in terms of product post-production and for customer relationship management, etc., was considered. Four measurement questions were specifically included to measure the customer demand management capability of the company.
5. The measurement of environmental competitiveness mainly reflects the intensity of competition, price competition, the situation of competitors, etc. The measurement scale was mainly based on the research results of Bengtsson and Sölvell (2004) and Claro et al. (2003). Three questions were included, such as “The price competition in our market is fierce.”
6. The measurement of supply chain financing performance mainly draws on the research results of Song and Lu (2017). It was measured through four aspects: financing amount, financing cost, financing cycle, and financing availability, and included four specific topics.

### Data collection

In this paper, data were collected using a seven-point Likert scale. Respondents rated the questions on a scale of 1–7 (from “strongly disagree” to “strongly agree”) according to the actual situation of the company. This paper focused on exploring what antecedent conditions affect the supply chain financing performance of SMEs. The main research target was to meet two conditions: first, to meet the “small and medium-sized enterprises classified as standard regulations” of small and medium-sized enterprises, mainly with reference to the provisions of the research enterprises in the industry, the size of enterprises, business assets, etc., to screen; second, the company was required to have experience in supply chain financing in the past year, including but not limited to warehouse receipt pledge financing, accounts receivable/payable pledge financing, inventory pledge financing, etc. The data collection for this time period mainly used online questionnaires. The online questionnaire was mainly distributed through the Credamo platform, and the questionnaire included two parts: first, the basic information of the enterprise, including the name of the enterprise, whether the enterprise has had supply chain financing experience in the past year, the scale of the enterprise, and other information; the second was a specific question item for variable measurement. A total of 250 questionnaires were distributed online. A total of 171 were returned, and 90 valid questionnaires were finally obtained through screening, with an efficiency rate of 52.6%. Some characteristics of the sample are listed in the Table 1 (The paper is based on a Chinese context and the data collected are based on companies in China).

**Table 1:** Structure of the sample distribution (*N* = 90).

Variable symbols	Variable meaning	Indicators	Number of frequency	Frequency (%)
EQ1	Enterprise size (Number of employees)	Less than 100 people	18	16.9%
		101–500 people	38	35.8%
		501–2,000 people	28	26.4%
		More than 2,000 people	6	5.6%
EQ2	Years of business operation	Less than 1 year	2	1.8%
		1–5 years	18	16.9%
		5–10 years	25	23.5%
		More than 10 years	45	42.4%
EQ3	Annual corporate sales	Under 1 million	6	5.6%
		1–10 million	28	26.4%
		10–50 million	24	22.6%
		50 million or more 5,000万以上	32	30.1%
EQ4	Total corporate assets	Under 1 million	8	7.5%
		1–10 million	14	13.2%
		10–50 million	22	20.7%
		50 million or more	46	43.3%

## Research method and data pre-processing

### Reasons for choosing the fsQCA

In this paper, we mainly applied the fsQCA method based on set theory. QCA was developed in the late 1980s by Charles C. Ragin in 1987, and takes a holistic view of comparative case-level analysis, with each case considered as a “grouping” of conditional variables (Rihoux and Ragin, 2009). The aim is to identify complex causal relationships between conditional groupings and outcomes through comparisons between cases. Based on technical tools such as ensemble and Boolean algebra, it aims to blend the advantages of qualitative and quantitative research methods. An attempt was made to analyze the multifaceted and complex relationships behind the supply chain financing performance of SMEs based on a histological perspective. This was mainly due to the following considerations: First, to derive the path to improve the performance of supply chain financing for SMEs, which requires exploring the degree of information technology application, enterprise information sharing capability, enterprise qualification, supply chain capability, environmental competitiveness, and other factors together, the method can break through the limitations of traditional regression analysis methods for overlapping effects among variables, and provide a systematic and comprehensive view to deal with the conformational problem of multiple interrelated factors acting on the results simultaneously; Second, fsQCA analysis suggests that the interdependence and different combinations of causal conditions can constitute multiple concurrent causal relationships, which contributes to a deeper understanding of the differential driving mechanisms of SME supply chain financing performance; Third, the qualitative comparative analysis of fuzzy sets is closer to reality than clear sets and multi-valued sets in terms of calibration, because it requires the data handled by continuous values to be between 0 and 1. In summary, this paper argues that the fsQCA approach is more suitable for exploring the mechanisms of the roles of many factors in the supply chain financing performance of SMEs from a holistic perspective.

### Reliability testing

Before conducting the fsQCA analysis, in order to ensure the high reliability and accuracy of the collected data, it was necessary to test the reliability and validity of the data, mainly with the help of software R for each antecedent variable. The supply chain capability at the organization level was mainly measured by the two indicators, the distribution operation capability and demand management capability, so the reliability of the two indicators is reported here. The results are shown in Table 2. The Cronbach’s α for the six antecedent variables and one outcome variable were all greater than 0.7, indicating that the question items had high reliability. The combination reliability (CR) values of the combined reliability of all variables were greater than 0.7, indicating the high internal consistency of the model. The average extracted variance (AVE) of each variable was higher than 0.5, indicating that the data scale had good convergent validity.

**Table 2:** Reliability test of each variable (*N* = 90).

Variable symbols	Antecedent conditions	Cronbach’s α	CR	AVE
IT	The Degree Of Application Of Information Technology	0.871	0.874	0.635
IS	Information Sharing Capability	0.869	0.871	0.692
EQ	Enterprise Qualification	0.874	0.886	0.668
DO	Distribution Operations Capability	0.926	0.927	0.680
RM	Demand Management Capability	0.844	0.846	0.580
EC	Environmental Competitiveness	0.874	0.876	0.639
SCP	Supply Chain Financing Performance	0.918	0.919	0.739

### Variable calibration

Calibration means the process of assigning a set affiliation score to a case (Schneider and Wagemann, 2012). In the qualitative comparative analysis of fuzzy sets, its requirement for the data to be processed is a continuous set between 0 and 1, called the degree of membership. After that, three anchor points are selected based on theoretical and practical experience, and the conditions and results of each case are classified as fully affiliated, semi-affiliated, and fully unaffiliated according to the selected anchor points. The calibration process in this paper was as follows: first, the scores of the measured question items of each variable were summed and averaged, and the mean value was used as the score of each variable to avoid the influence of too large a value of one variable on the whole data; secondly, the key to calibration lay in the choice of anchor points, although the Likert scale already differentiates between the degree and level of specific conditions (variables) in the design phase (Zhang and Du, 2019). However, since most of the surveyed companies belonged to a large scale and the knowledge level of the questionnaire respondents varied, there may be a large subjective will in scoring. Therefore, this paper drew on existing research and applied the direct calibration method on the basis of existing theoretical and empirical knowledge. Taking into account, the actual situation and drawing on the study of Fiss (2011), the three anchor points of the five conditional and outcome variables were set to corresponding values of 25% (fully unaffiliated), 50% (intersection), and 75% (fully affiliated), and the final calibration results are shown in Table 3.

**Table 3:** Calibration values for each variable.

Variable	IT	IS	EQ	SC	EC	SCP
Fully affiliated	6.75	6.67	3.89	6.66	6.50	6.75
Intersection	6.00	5.67	3.00	5.80	5.25	5.50
Fully unaffiliated	4.11	4.00	1.36	4.05	3.50	3.50

### Necessity analysis

Before the group analysis, the necessity of the results needed to be checked with respect to each antecedent condition. Necessity analysis refers to whether the appearance of a certain result is necessarily accompanied by the appearance of a certain condition. However, the occurrence of that condition does not necessarily lead to the occurrence of that result. In general, the minimum criterion for determining the necessary condition is greater than 0.9. As shown in Table 4, no single conditional variable had an effect on high/low supply chain financing performance that exceeded 0.9, that is, the results suggest that no single factor can constitute a necessary condition for high or low supply chain financing performance. This shows that the variation in the performance of firms’ supply chain financing is the result of a combination of multiple factors.

**Table 4:** Results of necessity tests for individual conditions.

Conditional variables	Result Variables	
	High supply chain financing performance	Low supply chain financing performance
**IT**	0.826	0.567
**~IT**	0.484	0.826
**IS**	0.819	0.617
**~IS**	0.496	0.782
**EQ**	0.709	0.629
**~EQ**	0.530	0.674
**SC**	0.868	0.629
**~SC**	0.496	0.832
**EC**	0.713	0.637
**~EC**	0.571	0.722

## Research findings

### Construction of truth table

After the necessity analysis of each antecedent condition, the data needed to be imported into the fsQCA 3.0 software for the construction of the truth table. The truth table mainly reflects the distribution of different sets of antecedent condition combinations on the results. The truth table gives a clear idea of how many antecedent condition combinations produce high/low supply chain financing performance, as well as the logical residuals. The key to the construction of the truth table is the selection of the case frequency threshold and the determination of the original consistency. For the selection of the case frequency threshold, the suggestion of Ragin (Rihoux and Ragin, 2009) was referred to: when the sample size is small, set the frequency to 1; additionally, the frequency threshold should be selected to retain at least 75% of the total number of cases. In terms of the original consistency threshold setting, Ragin recommends a corresponding minimum threshold of 0.8. In summary, in this paper, the case frequency was set to 1, the original consistency threshold was set at 0.8, and the PRI consistency was set to 0.7. Different groups of high supply chain financing performance and non-high supply chain financing performance were generated in the form of 0 and 1.

### Configuration analysis

Unlike the analysis of necessary conditions, group analysis attempts to reveal the sufficiency of different groupings consisting of multiple conditions to cause the generation of results (Tao et al., 2021). When the truth table is constructed, the software performs operations and then generates three solutions (complex, intermediate, and parsimonious). Since the intermediate solution is produced using an easy counterfactual analysis that includes core and edge conditions for the grouping, the results are more likely to reflect the actual results. In addition, given that existing studies generally report intermediate solutions, this paper mainly reports the intermediate solution. The results of the standardized analysis of high/low supply chain financing performance are shown in Table 5. It can be seen that there were three antecedent condition groups that generated high supply chain financing performance, while there was only one antecedent condition group that generated low supply chain financing performance. Among them, the consistency values of the high supply chain financing performance group (Z1, Z2, and Z3) were 0.931, 0.969, and 0.963, and the consistency of their overall solutions was 0.928. It is shown that these three conditions were sufficient to generate high supply chain financing performance. The overall solution coverage was 0.779, indicating that these three conditional groupings explained 77.9% of the cases. The consistency value was 0.927 for the low supply chain financing performance grouping (Z4) and 0.927 for the overall solution. The overall solution coverage was 0.543, indicating that the article grouping was able to explain 54.3% of the cases. The results of these antecedent condition groupings suggest that the degree of information technology application, information sharing capability, firm qualification, and supply chain capability, and the presence or absence of a competitive external environment can lead to differences in the supply chain financing performance of SMEs.

**Table 5:** Group analysis of high and low supply chain financing performance.

Variable category		A grouping that generates high supply chain financing performance			Generating low supply chain finance performance groups
		Z1	Z2	Z3	Z4
Technical level	**IT**	●	●		ⓧ
	**IS**	●		●	ⓧ
Organizational level	**EQ**		●	●	
	**SC**	●	●	●	ⓧ
Environmental level	**EC**		ⓧ	ⓧ	ⓧ
Consistency		0.931	0.969	0.963	0.927
Unique coverage		0.334	0.036	0.034	0.543
Original coverage		0.708	0.410	0.409	0.543
Result coverage		0.779			0.543
Results Consistency		0.928			0.927

Referring to the formulation of Ragin (Claro et al., 2003), ● indicates that the variable exists, ⓧ indicates that the variable does not exist among them. Large circles denote core conditions, small circles denote edge conditions, and a blank space indicates that the presence or absence of the variable is irrelevant.

### Path analysis

#### The conditional grouping of high supply chain financing performance

From Table 5, it can be seen that the three grouping paths leading to high supply chain financing performance were different and can be considered as a sufficient condition grouping for high supply chain financing performance.

Configuration Z1: the degree of application of information technology * information sharing capability * supply chain capability. The path suggests that the presence of these three conditions plays a central role for SMEs to generate high supply chain financing performance that can break through the environmental conditions. Regarding the research topic of supply chain financing, companies with high supply chain financing performance invest more resources and incorporate technology level activities as a way to improve their information technology and keep up with the developments of the times. The information-sharing ability of enterprises can better solve the problem of information asymmetry among supply chain enterprises through a high degree of information technology application, thus making the core enterprises in the supply chain more aware of the operation of enterprises. In addition, the existence of supply chain capabilities indicates that the company has a greater advantage in distribution operations and the management of customer demand, can signal to the outside world that the company has stable operating profits, and can increase the willingness of core firms to provide supply chain financing solutions for them, which in turn helps SMEs to obtain higher supply chain financing performance. For example, Tongchuang Mastery Technology Co. Without regard to corporate qualifications and competitive pressures in the external environment, the company has expanded inward and focused on the development of its own technical capabilities, with integrity being its core business. Further, the company is deeply committed to the improvement of corporate supply chain capabilities in the development process, focusing on the growth and development of corporate partners, which ultimately manifests itself in high supply chain financing performance. As this driving path is composed of the degree of IT adoption, information sharing capabilities (technology), and supply chain capabilities (organization), in this paper, we named it “technology-supply chain capability “. The consistency of this grouping was 0.931, the unique coverage was 0.334, and the original coverage was 0.708. This path can explain about 70.8% of the corporate supply chain financing cases. In addition, about 33.4% of corporate supply chain financing cases can be explained by this path only.

Configuration Z2: The degree of application of information technology * enterprise qualification * supply chain capability * ~ environmental competitiveness. This shows that SMEs have certain strengths when they have a higher degree of IT application and a stronger supply chain capability, and when faced with weaker competition from the external environment, firms are still able to achieve higher supply chain financing performance regardless of whether they have high or low information-sharing capabilities. Guangdong Wanfang Construction Co. can be taken as an example: although its size is small and it is under certain competitive pressures from the external environment, it still presents a high supply chain financing performance, because of its strong information technology utilization and supply chain capabilities, which stand out from the crowd. The consistency of this grouping was 0.969, the unique coverage was 0.036, and the original coverage was 0.410. This path explained about 41% of corporate supply chain financing cases, but only 3.6% of corporate supply chain financing cases can be explained by this path only.

Configuration Z3: information sharing capability * corporate qualification * supply chain capability * ~ environmental competitiveness. Among them, the presence of two antecedent conditions, information sharing capability, and supply chain capability, plays a central role in generating high supply chain financing performance for firms, and the presence of firm qualifications plays a supporting role. Liaoning Photoelectric Technology Co. can be taken as an example of this. Despite the company’s shortcomings in the use of information technology, the company has been able to communicate and cooperate frequently with its partners, increasing mutual understanding and trust, and has been able to focus on improving its own information-sharing ability and supply chain capability to increase the willingness of core companies to guarantee them, ultimately helping them obtain a high supply chain financing performance. The consistency of this grouping was 0.963, the unique coverage was 0.034, and the original coverage was 0.409. This path explained about 40.9% of corporate supply chain financing cases, but only 3.4% of the sample cases can be explained by this path only.

By comparing the above high supply chain financing performance condition groupings (Z1, Z2, and Z3), it was found that supply chain capability appeared in each path as a core condition, indicating that strong supply chain capability is a strong driver for SMEs to generate high supply chain financing performance. Secondly, by comparing Z2 with Z3, it was found that there is a substitution between IT application and information sharing capability when SMEs have strong supply chain capability. In other words, when SMEs have distribution operation capability and customer demand management capability, they can present high supply chain financing performance when they reach a high level of information technology application or meet the information-sharing capability requirement. Finally, by comparing Z1 with Z2 and Z1 with Z3, it was found that, when SMEs face weaker competitive pressures and have a better survival environment, SMEs with certain strengths will obtain high supply chain financing performance after mastering supply chain capabilities, provided that one of the technical-level conditions is met.

#### Conditional grouping of low supply chain financing performance

Configuration Z4:~Information technology application degree* ~ Information sharing capability* ~ Supply chain capability* ~ Environmental competitiveness. When firms face weaker competition in the external environment, a low level of utilization of information technology, as well as a lower information sharing capability and lower supply chain capability, will inhibit the performance of supply chain financing for SMEs, regardless of the strength of the company. For example, Suzhou Centennial Legend Food Co. This company belongs to a traditional food company, which does not pay enough attention to the application of information technology and the development of corporate supply chain capabilities, and it is difficult for this company to be identified by core companies, thus generating a lower supply chain financing performance. Among them, the degree of information technology utilization and information sharing capability belongs to the technical level, the supply chain capability belongs to the organizational level factor, and the competitiveness of the external environment belongs to the environmental level factor. This triple dimension has a synergistic inhibitory effect on the supply chain financing performance of SMEs. This path explained 54.3% of the low supply chain financing performance sample among SMEs, and also 54.3% was explained by this path.

In summary, supply chain capability appeared as a core variable in Z1, Z2, and Z3, which had high supply chain financing performance, while a lack of supply chain capability was missing as a core variable in the Z4 group state, which had low supply chain financing performance. Second, both IT application and information sharing capability appeared as core variables in Z2 and Z3, respectively, while both were missing as core variables in Z4. Therefore, it can be determined that the application of corporate information technology, information sharing capability, and supply chain capability has a significant positive effect on the ability of SMEs to generate high supply chain financing performance.

### Robustness tests

In the fsQCA method, the robustness test is a crucial part to check whether the condition set is stable and can guide the enterprise practice. This paper focused on robustness testing by adjusting the original consistency and PRI consistency, as well as the case frequency thresholds. First, the original consistency threshold of 0.80 was increased to 0.85, using a test with a variable consistency threshold. Second, the PRI consistency was increased from 0.7 to 0.75. Finally, the case frequency threshold was adjusted by increasing the original threshold from 1 to 2. The three methods produced consistent groupings, it can be concluded that the findings of this paper were robust.

## Discussion

### Theoretical contribution

First, the research enriches the study of factors influencing the performance of corporate supply chain financing, as most of the existing studies are based on a single variable or are bivariate studies on the impact of supply chain financing performance. Then, when verifying the correlations between variables, the research perspectives are generally more homogeneous. A deeper analysis of the reasons affecting the supply chain financing performance of SMEs is needed, as well as enriching the scope of supply chain financing performance research. This paper attempted to analyze how the three dimensions of technology, organization, and environment interact with each other in SME supply chain financing performance based on the TOE research framework, as well as how they work together in SME supply chain financing performance.

Second, this research was process-oriented in order to increase the practicality of performance research. The research formed the path of high/low supply chain financing for SMEs, which helps to enhance the practicality of supply chain financing performance, and is more instructive for companies. Is the path to forming a firm’s high supply chain financing performance is unique? and can SMEs choose a high supply chain financing performance path that suits their own development according to their actual situation? Further, what are the differences between the paths that lead to high supply chain financing performance and those that lead to low supply chain financing performance? Do the antecedent conditions in the pathway have a substitution relationship with each other? Studying the factors influencing the performance of low supply chain financing is more relevant for companies.

### Practical enlightenment

First, SMEs should pay high attention to the issue of variability and diversity in the grouping of supply chain financing performance. There is a linkage matching effect between the various influencing factors. Therefore, in order to obtain supply chain financing provided by core enterprises and solve their own financing constraints, enterprises should pay attention to the synergistic development of multiple links of enterprises. Since SMEs in the supply chain are in different situations and face different external competitive environments, the causes of low supply chain financing performance should be determined according to the actual situation and the right remedy should be prescribed so as to effectively improve supply chain financing performance.

Secondly, SMEs should attach great importance to the development of corporate capabilities. In the supply chain financing model, the various capabilities of SMEs influence the willingness of core enterprises to provide credit guarantees, which in turn affects whether SMEs can obtain credit support from financial institutions. Therefore, the capacity of SMEs is particularly important and critical. Furthermore, the analysis of the above grouping results shows that the supply chain capability of firms plays a key role in improving the supply chain financing performance of SMEs. SMEs should pay attention to the development of their own supply chain capabilities so that they can be recognized by core enterprises or financial institutions and increase the availability of supply chain financing, thus effectively improving supply chain financing performance.

## Conclusion and outlook

### Conclusion

In this paper, based on the TOE framework model, a sample of 90 SMEs involved in the supply chain financing business was studied. Using the fsQCA method, the grouping paths that generate high versus low supply chain financing performance of SMEs were analyzed to explore the grouping paths of conditional variables affecting supply chain financing performance at three levels. Three grouping paths with high supply chain financing performance, and one with low supply chain financing performance were generated. The main findings are as follows.

First, through a single-condition necessity analysis, it was found that none of the five factors in the three dimensions of “technology + organization + environment” is necessary to produce high supply chain financing performance. Among them, there are three different models of high supply chain financing performance: “technology-supply chain capability-driven,” “IT-supply chain capability-driven,” and “IS-supply chain capability-driven.” These three models are driving the performance of SME supply chain financing in “different routes to the same destination.”

Second, the two antecedent conditions at the technical level are interchangeable. This implies that the application of corporate information technology and the ability to share information can be interchangeable in a given situation, producing an equivalent effect of effectively improving the supply chain financing performance of SMEs. In addition, the application of corporate information technology and the ability to share information and supply chain capabilities play a critical role in facilitating the performance of supply chain financing for SMEs.

### Research limitations and outlook

The pathway of the role of SME supply chain financing performance derived in this paper has the following limitations, which need to be further explored in subsequent studies: (1) Based on the existing research results, this paper constructed a TOE analysis model with three levels and five conditional variables, without considering the influence of other factors on the performance of supply chain financing. However, the possibility of the existence of other factors or groupings that have a role in the performance of supply chain financing cannot be excluded. (2) The cases studied in this paper were 90 SMEs identified after screening, and further research is needed to determine whether the findings are generalizable to all SMEs. (3) The data of this paper were obtained from the questionnaire survey. Due to the uneven quality of the respondents, the understanding of the questionnaire items was influenced by subjective will, which to a certain extent affects the quality of the conclusions obtained in this paper.

With the continued decay of the global economy, the heavy pressure on SMEs. The procurement phase, the rise in raw material prices, further led to the increase in production costs of SMEs. Production stage, along with the continued impact of the epidemic, some areas are still in the stage of shutdown, enterprise plant rent, equipment depreciation, personnel wages, continued to compress the survival of small and medium-sized enterprises. During the sales phase, the domestic market is shrinking, people are afraid to consume, and enterprises cannot collect funds, leading to serious cash flow break problems for many enterprises. At this time, enterprises should establish the concept of sustainable development, accelerate the digital transformation of enterprises, shrink production lines, specialize in the strengths of the enterprise products, digital production, improve production capacity, retain cash flow, and live as the primary strategy of the enterprise. Future research on the topic of supply chain finance could focus on whether the efforts made by SMEs on sustainability will increase the willingness of core firms in the supply chain to guarantee them and thus improve the supply chain financing performance of SMEs. And how supply chain finance can empower the digital transformation of enterprises.

## Data availability statement

The raw data supporting the conclusions of this article will be made available by the authors, without undue reservation.

## Author contributions

HH: methodology, software, investigation, data curation, writing—original draft preparation, and visualization. HH and YZ: validation. YZ: formal analysis and project administration. WD and YZ: resources and writing—review and editing. WD: supervision. All authors contributed to the article and approved the submitted version.

## Funding

This study was supported by the National Social Science Foundation of China “Influencing Factors of Liability of Foreignness in the Internationalization of Chinese Firms” (No. 19BGL024).

## Conflict of interest

The authors declare that the research was conducted in the absence of any commercial or financial relationships that could be construed as a potential conflict of interest.

## Publisher’s note

All claims expressed in this article are solely those of the authors and do not necessarily represent those of their affiliated organizations, or those of the publisher, the editors and the reviewers. Any product that may be evaluated in this article, or claim that may be made by its manufacturer, is not guaranteed or endorsed by the publisher.
